# New Frontiers in Caregiving Research: Biopsychosocial Perspectives and Interventions

**DOI:** 10.1093/geroni/igaa057.2086

**Published:** 2020-12-16

**Authors:** Jyoti Savla, Karen Roberto, Mamta Sapra

**Affiliations:** 1 Virginia Tech, Blacksburg, Virginia, United States; 2 Salem VA Medical Center, Salem, Virginia, United States

## Abstract

Although families embrace the opportunity to care for a loved one, caregiving is stressful and takes a toll on the caregiver’s health and well-being. Earlier studies of stress and coping among family caregivers focused on psychological outcomes and emotional well-being. In the last decade, stress researchers have broadened their focus to include biomarkers and health outcomes. Data from two studies of caregivers of persons with memory loss will be used to discuss two new frontiers of caregiving research. First, a daily-diary study will be used to identify the mechanism by which stress disrupts the physiological processes and proliferates into serious psychopathology and pre-clinical and clinical health conditions. Second, a mindfulness-based psychoeducational intervention study will be utilized to identify malleable factors that can be harnessed to lower stress and improve the well-being of family caregivers. Next steps for caregiving research in the context of demographic and technological trends will be discussed.

